# Culture as a Moderator of Epistemically Suspect Beliefs

**DOI:** 10.3389/fpsyg.2022.745580

**Published:** 2022-02-09

**Authors:** Yoshimasa Majima, Alexander C. Walker, Martin Harry Turpin, Jonathan A. Fugelsang

**Affiliations:** ^1^Department of Psychology for Well-Being, Hokusei Gakuen University, Sapporo, Japan; ^2^Department of Psychology, University of Waterloo, Waterloo, ON, Canada

**Keywords:** epistemically suspect beliefs, cultural differences, analytic thinking, analytic-holistic cognition, bullshit receptivity

## Abstract

A consistent finding reported in the literature is that epistemically suspect beliefs (e.g., paranormal beliefs) are less frequently endorsed by individuals with a greater tendency to think analytically. However, these results have been observed predominantly in Western participants. In the present work, we explore various individual differences known to predict epistemically suspect beliefs across both Western and Eastern cultures. Across four studies with Japanese (*n* = 666) and Western (*n* = 650) individuals, we find that the association between thinking style and beliefs varied as a function of culture. Specifically, while Westerners who scored higher on measures of Type-2 analytic thinking tended to endorse epistemically suspect beliefs less, this association was not observed in Japanese samples, suggesting that the often-observed negative association between analytic thinking and epistemically suspect beliefs may be exclusive to Western individuals. Additionally, we demonstrate that a tendency to think holistically (specifically with regards to causality) is positively associated with the endorsement of epistemically suspect beliefs within both samples. Overall, we discuss how various individual differences predict the endorsement of epistemically suspect beliefs across cultures.

## Introduction

Fundamentally, scientists are in the business of trying to improve the accuracy of both their own and humanity’s beliefs via the collection of information about the universe. As such, it is no surprise that a community of researchers have become profoundly interested in epistemically suspect beliefs (ESBs), which refer to beliefs that do not cohere with established scientific evidence (e.g., paranormal beliefs; Lobato et al., 2014). Previous studies investigating ESBs have focused on the individual differences of believers as opposed to skeptics, including differences in cognitive ability, reasoning skills, and thinking style. These findings suggest that those who endorse ESBs are in general less educated (Gray and Mill, 1990; Aarnio and Lindeman, 2005), perform worse on some reasoning tasks (Blackmore and Trościanko, 1985; Roberts and Seager, 1999), and engage less in analytic thinking compared to skeptics (Lindeman and Aarnio, 2006; Pennycook et al., 2012).

Analytic thinking in this context refers to thought processes that are commonly characterized as being deliberative, reflective, and requiring working memory (i.e., Type-2 processes; Evans, 2008; Evans and Stanovich, 2013). Much research has shown that analytic thinking is a good predictor of performance on a range of reasoning and decision making tasks (e.g., heuristics-and-biases tasks that involve successfully overriding an incorrect intuitive response; Toplak et al., 2011). With regard to the endorsement of everyday irrational beliefs, such as superstitions, research has suggested that these beliefs arise from our intuitive processes (i.e., Type-1 processes; Evans, 2008; Evans and Stanovich, 2013), tend to rely on heuristics, seek for coherent causal explanations, and favor evidence providing supports for one’s current beliefs (Risen, 2016). Therefore, a line of argumentation like ESBs providing simple surface-level explanations about the universe may be intuitively appealing. These beliefs will be maintained unless they are more closely re-examined by analytic thinking. In line with this claim, other work has found that the tendency to engage in analytic thinking is negatively associated with various forms of ESBs including religious belief (Gervais and Norenzayan, 2012; Pennycook et al., 2012, 2014), belief in the paranormal (Aarnio and Lindeman, 2005; Lobato et al., 2014), and pseudoscientific beliefs (Lindeman, 2011; Lobato et al., 2014). Relatedly, analytic thinking has been shown to be negatively associated with receptivity to superficially impressive yet vacuous statements (i.e., pseudo-profound bullshit; Pennycook et al., 2015a; Walker et al., 2019). On the basis of these findings, many scholars have argued that ESBs are rooted in Type-1 intuitive processing which can be overridden by effortful and Type-2 analytic processes (Pennycook et al., 2015b).

Although the negative association between ESBs and analytic thinking appears robust, it may be reasonable to be skeptical about the underlying mechanisms proposed. First, in the domain of religious belief, studies have shown contradictory findings. For example, the role of analytic thinking in supporting religious disbelief has been challenged by research showing that promoting analytical thinking does not promote religious disbelief (Yonker et al., 2016; Sanchez et al., 2017). Furthermore, most studies examining the association between cognitive style and ESBs have been conducted exclusively with WEIRD (Western, Educated, Industrialized, Rich and Democratic; Henrich et al., 2010) participants. Therefore, it remains an open question whether the link between analytic thinking and ESBs generalizes to non-WEIRD populations. It is important to examine possible cultural differences in the underlying processes associated with ESBs in order to better understand everyday irrational beliefs. If cultural differences in thinking styles are identified between populations, they are likely to interact with interventions focused on thinking (e.g., education, and debiasing). Furthermore, cross-cultural comparisons of everyday beliefs are important as they can lead to a better understanding of cultural differences on the effects of various psycho-social factors on well-being (Yong et al., 2021).

Recent work has begun to investigate cross-cultural differences as they relate to ESBs. For example, compared to Westerners, Chinese individuals have been found to be more likely to endorse paranormal beliefs (Shiah et al., 2010) and Turkish individuals more likely to endorse conspiracy beliefs (Bruder et al., 2013). Relatedly, Bahçekapili and Yilmaz (2017) reported a series of studies featuring Muslim populations showing that analytic thinking was negatively associated with intrinsic/extrinsic motivations for religiosity (e.g., personal duty or societal pressure), but positively associated with religiosity dealing with an open-minded seeking of answers to existential questions. Based on these findings, Bahçekapili and Yilmaz (2017) suggest that the link between analytic thinking and religiosity depends on how religiosity is expressed among individuals. Relatedly, Tosyali and Aktas (2021) show that the negative link between analytic thinking and superstitious beliefs is stronger for Turkish participants with low-to-moderate levels of religiosity than highly religious individuals. These results suggest that the relationship between analytic thinking and irrational beliefs is not as simple as analytic thinking always suppressing such beliefs, but may involve group differences related to norms surrounding belief, such as culture. In the domain of paranormal and pseudoscientific belief, Japanese individuals self-reporting a strong tendency toward analytic thinking, measured by the rationality subscale of the Rational-Experiential Inventory (Pacini and Epstein, 1999), were more likely to hold paranormal and pseudoscientific beliefs (Karasawa and Tsukimoto, 2010; Majima, 2015), providing initial evidence that the commonly observed negative association between analytic thinking and ESBs may not generalize to non-WEIRD populations. Nevertheless, participants’ level of analytic thinking was self-reported within this study, leaving open the possibility that they were simply mis-calibrated in their self-assessment. Conversely, it could be that the link between analytic thinking and ESBs is absent in Japanese samples, perhaps on account of ESBs being less in violation of Japanese as opposed to Western cultural norms.

Along with differences in ESBs, findings from cultural psychology have demonstrated differences in cognitive style between Western and Eastern populations. These findings suggest that Westerners are more likely to adopt ‘analytic’ modes of cognition, while Easterners are more likely to take holistic approaches (Nisbett et al., 2001; Nisbett and Miyamoto, 2005; see also Buchtel and Norenzayan, 2009 for discussions regarding differences between Type-2 analytic thinking as described by contemporary dual process theorists and ‘analytic’ cognition as described in the domain of cultural psychology). Generally speaking, in the domain of cultural psychology, ‘analytic’ individuals tend to focus on the specific attributes or elements of an object or problem, rather than the larger context as a whole. In contrast, rather than focusing on individual elements, holistic individuals tend to focus on the totality of an object or problem, including the overarching context. Furthermore, holistic thinking has been shown to predict the acceptance of mutually conflicting statements (naïve dialecticism; Spencer-Rodgers et al., 2010) and complex causality (Maddux and Yuki, 2006). Relatedly, past work demonstrates that Eastern (i.e., Japanese) participants tend to report more mixed emotions than Americans, predominantly in pleasant situations (Miyamoto et al., 2010).

These findings suggest that differences in the endorsement of ESBs across Western and Eastern cultures may be explained, at least partially, by cultural differences in analytic-holistic modes of thinking. Since holistic cognition has a more dialectical orientation, such as the acceptance of contradiction and complex causation, it is reasonable to assume that holistic thinkers may be more likely to accept mutually conflicting statements and as a result show greater endorsement of ESBs than those engaging in more rule-based, or ‘analytic’ modes of reasoning. Therefore, the tendency for Eastern individuals to think holistically may offer one explanation for why Eastern individuals seemingly endorse more ESBs compared to Western individuals. However, readers may by puzzled by the distinction between Type-2 analytic thinking, as discussed by dual process theorists, and an ‘analytic’ mode of cognition, as discussed by cross-cultural psychologists. For this difference, Buchtel and Norenzayan (2009) argue that Type-2 analytic thinking and cultural ‘analytic’ cognition share similarities with regards to context independence and weak attention to social relations. Nevertheless, holistic cognition does not necessarily correspond to Type-1 processes, nor does ‘analytic’ cognition necessarily correspond to Type-2 processes. Rather, Buchtel and Norenzayan (2009) posit that analytic-holistic modes of thinking can be viewed as different styles, or individual variations of thinking, that operate under the umbrella of Type-2 processes. From this perspective, the negative association between so-called Type-2 analytic thinking and ESBs may not necessarily be culturally universal.

The present research investigates endorsement of ESBs (e.g., paranormal and pseudoscientific beliefs) within samples of Western (North American and Western European) and Eastern (Japanese) participants. We seek to not only assess the frequency of ESBs across Western and Eastern populations, but also investigate whether such beliefs are predicted by the same individual difference variables (e.g., Type-2 analytic thinking) across cultures. Based on past work (Shiah et al., 2010; Bruder et al., 2013), we expect Western participants to endorse ESBs less than Eastern participants. We examine whether such a difference can be explained by Western participants preferring more ‘analytic’ and linear styles of thinking compared to Eastern participants, who more frequently engage in holistic and dialectic styles of thinking (Nisbett et al., 2001; Nisbett and Miyamoto, 2005).

Lastly, we investigate receptiveness to pseudo-profound bullshit across Western and Eastern cultures. Similar to holding ESBs, endorsement of pseudo-profound bullshit (i.e., statements that are superficially impressive yet consist of a largely random assortment of profound-sounding words) has been argued to result from a failure to engage Type-2 analytic thinking (Pennycook et al., 2015a; Pennycook and Rand, 2020). That is, endorsement of pseudo-profound bullshit shares a common cognitive mechanism with acceptance of ESBs. Relatedly, studies have demonstrated positive associations between bullshit receptivity and real-world beliefs, such as the endorsement of paranormal beliefs (Pennycook et al., 2015a) and “fake news” (Pennycook and Rand, 2020). Nevertheless, the claim that bullshit receptivity naturally follows from a failure to engage Type-2 analytic thinking may be incomplete as other factors such as the tendency to perceive patterns or maintain radically subjective beliefs may similarly explain receptiveness to pseudo-profound bullshit statements (Turpin et al., 2019; Walker et al., 2019). These factors likely vary based on culture and so too may receptiveness to pseudo-profound bullshit.

## Ethics Statement

All studies were conducted in accordance with APA ethical standards and approved by the relevant ethics committees. All individuals gave their informed consent online prior to participation.

## Study 1

### Method

#### Participants

A sample of 298 participants were recruited from two online crowdsourcing platforms, 147 Japanese participants (59% female; *M*_age_ = 38.37, *SD*_age_ = 9.45) from CrowdWorks (CW) and 151 North American and European participants (47% female; *M*_age_ = 33.23, *SD*_age_ = 11.50; 36% United States residents, 38% United Kingdom residents, 25% other) from Prolific Academic (ProA)^1^. All participants received compensation (CW = 240 JPY; ProA = £2.00) upon completion of an online questionnaire. For all studies, participants were required to possess an approval rating of 95% or higher on either CW or ProA in order to be eligible to participate. We collected our full sample prior to data analyses, report all data exclusions, all manipulations, and all measures used.

#### Materials

##### Paranormal Belief Scale

We assessed the degree to which participants endorsed various paranormal beliefs by asking them to judge the plausibility of 12 paranormal belief items (B_PA_). These items were drawn from the Psi questionnaire (6-items; Roberts and Seager, 1999; Japanese version adopted from Majima, 2015) and Revised Paranormal Belief Scale (6-items; adopted from Tobacyk, 2004^2^). Participants judged the plausibility of each B_PA_ item (e.g., “How likely is it that you possess some form of ‘psychic ability’?”) using a five-point scale ranging from 1 (*Extremely Unlikely*) to 5 (*Certain*). Ratings given to each item were averaged to calculate a B_PA_ score for each participant. A complete list of items (for all studies) can be viewed in the Supplementary materials.

##### Pseudoscientific Belief Scale

We assessed the degree to which participants endorsed pseudoscientific beliefs with 12 pseudoscientific belief (B_PS_) items (e.g., “Homeopathic remedies foster spontaneous healing”). This scale consisted of six items from Majima (2015), three items from Lobato et al. (2014), and three items from Dekker et al. (2012)^3^. Participants were asked to judge their agreement with each item on a five-point scale ranging from 1 (*Disagree)* to 5 (*Agree*). Once again, ratings given to each item were averaged to obtain a B_PS_ score for each participant.

##### Cognitive Style Measures

We assessed participants’ tendency to engage in rational (analytic) and experiential (intuitive) thinking with 12 items drawn from Naito et al.’s (2004) Information-Processing Style Inventory (IPS). The original IPS contains 24 items adapted from Pacini and Epstein (1999) Rational-Experiential Inventory (REI), consisting of six items from each of the four REI subscales (rational engagement, rational ability, experiential engagement, and experiential ability). In order to reduce participants’ work load, we chose 12 items (three items from each subscale) showing high factor loading scores in studies with Western (Pacini and Epstein, 1999) and Japanese (Naito et al., 2004) participants. For each item, participants were presented with a statement and asked to judge the extent to which the statement was true of themselves on a five-point scale (1 = *Definitely not true*, 5 = *Definitely true*). Responses to six rationality items (e.g., “I enjoy intellectual challenges”) and six experiential items (e.g., “I like to rely on my intuitive impressions”) were averaged to obtain a rationality and experientiality score for each participant.

Participants were also presented with a three item Cognitive Reflection Test (CRT; Frederick, 2005), designed to provide a behavioral measure of participants’ ability to suppress an intuitive incorrect response in favor of an analytical correct answer. For all CRT items participants provided their answers in a free-entry text box. These two scales were introduced to measure individual differences in Type-2 analytic (rationality and CRT) and Type-1 intuitive (experientiality) thinking.

Lastly, we administered the Analysis-Holism scale (AHS; Choi et al., 2007) to assess participants’ tendencies to engage in analytic-holistic modes of thinking. The original AHS scale consisted of 24 items evenly split into four subscales (causality, attitude toward contradiction, perceived change, and locus of attention). However, to reduce participants’ work load, we only administered 12 AHS items (3 items from each subscale). Participants were presented each item individually and responded using a seven-point scale ranging from 1 (*Strongly disagree*) to 7 (*Strongly agree*). Within each subscale, ratings given to each item were averaged to obtain an AHS subscale score.

##### Cognitive Ability Measures

We assessed participants’ cognitive ability with a syllogistic reasoning (cf. Majima, 2015) and numeracy task. We measured participants’ logical reasoning ability using a syllogistic reasoning task adopted from Markovits and Nantel (1989). The syllogistic reasoning task presented participants with eight syllogisms, all of which featured a conflict between the logical validity of the syllogism and the believability of its conclusion. Importantly, this design ensured that for each item the intuitive response (based on conclusion believability) would need to be overridden in order for the correct answer to be produced. For each syllogism, participants were asked to indicate whether the conclusion followed logically from the premises presented, irrespective of the believability of the concluding sentence. The sum of correctly solved syllogisms was used as an index of the logical reasoning ability of each participant. Additionally, we measured participants’ numeracy using the Subjective Numeracy Scale (SNS; Fagerlin et al., 2007). This scale was developed and validated as a conventional self-evaluation numeracy scale. We computed an unweighted mean of Percent of Maximum Possible (POMP; Cohen et al., 1999) scores for both our syllogistic reasoning and numeracy task in order to obtain a joint measure of cognitive ability (CAB).

#### Procedure

Participants were administered an online questionnaire in which they were asked to complete several tasks in the following order: pseudoscientific belief items, paranormal belief items, information-processing style inventory items (we refer to this scale as the REI hereafter referencing the origin of these items), a syllogistic reasoning task, the SNS, the AHS, and the CRT. Following completion of these tasks participants were asked a series of demographic questions (i.e., age, gender, nationality, ethnicity, native language, and highest educational level). Among these questions, only age and gender were considered predictors for the subsequent analysis^4^. All materials were presented in Japanese for CW participants and in English for ProA participants.

### Results and Discussion

Table 1 displays the descriptive statistics and bivariate correlations for all key variables. With the exception of AHS subscales (α ranged from .60 to .75)^5^, all scales showed good internal consistency (α ranged from .79 to .94). A series of independent samples *t*-tests found that our two samples did not differ in cognitive ability, CRT performance, or experientiality scores (all *p*s > .10). However, Westerners scored higher on our measure of rationality (i.e., the six rationality subscale items included in the REI), *t*(296) = 6.22, *p* < .001, *d* = 0.72. Consistent with past work, Japanese participants endorsed more paranormal, *t*(296) = 5.81, *p* < .001, *d* = 0.66, and pseudoscientific beliefs, *t*(296) = 4.81, *p* < .001, *d* = 0.56, than Westerners. They also scored higher in three of the four AHS subscales: causality [*t*(296) = 3.09, *p* = .002, *d* = 0.36], perception of change [*t*(296) = 3.00, *p* = .003, *d* = 0.35], and locus of attention [*t*(290) = 5.92, *p* < .001, *d* = 0.69]. Surprisingly, no difference between samples was observed for the attitude toward contradiction AHS subscale, *t*(296) = 1.02, *p* = .307, *d* = 0.12.

**TABLE 1:** Descriptive statistics and correlation of key variables (Study 1).

		West (*n* = 151)		JP (*n* = 147)		Sample difference			Pearson correlational coefficients ^a^										
	α	*M*	*SD*	*M*	*SD*	*t*	*p*	*d*	1.	2.	3.	4.	5.	6.	7.	8.	9.	10.	11.
1. Age		33.23	11.50	38.37	9.45	4.22	<.001	0.49		−.003	.023	−.001	.029	.014	.030	.081	−.044	−.010	**.199**
2. CAB		66.62	21.10	62.95	18.04	1.61	.108	0.19	**.160**		**.305**	**−.274**	**.490**	.018	.015	−.040	.002	**−.215**	**−.165**
3. RAT	0.87	3.80	0.79	3.20	0.88	6.22	<.001	0.72	−.063	**.453**		**−.259**	**.177**	.071	.102	.011	**.235**	−.084	−.106
4. EXP	0.88	3.18	0.93	3.04	0.77	1.44	.151	0.17	.033	**−.285**	−.125		**−.299**	.029	**−.201**	−.139	−.147	**.481**	**.372**
5. CRT		1.63	1.18	1.44	1.09	1.47	.143	0.17	.098	**.646**	**.342**	**−.335**		.033	−.048	.097	.051	**−.255**	**−.273**
6. Cause	0.75	4.89	1.37	5.33	1.06	−3.09	.002	0.36	.054	−.151	−.066	**.221**	**−.169**		.053	−.057	**.195**	.081	.133
7. Contra	0.66	4.98	1.22	4.84	1.12	1.02	.307	0.12	−.031	−.104	−.068	**.178**	−.137	.015		−.035	**.206**	−.079	.121
8. Change	0.60	4.74	1.14	5.15	1.18	−3.00	.003	0.35	−.017	−.066	.016	.019	.079	**−.162**	.003		.014	**−.199**	−.161
9. Attention	0.64	4.51	1.30	5.28	0.91	−5.92	<.001	0.69	.001	.017	−.062	.024	−.052	.028	.097	−.075		−.045	−.069
10. B_PA_	0.94	2.24	1.03	2.87	0.83	−5.81	<.001	0.66	.126	**−.389**	**−.192**	**.393**	**−.486**	**.283**	.112	−.121	−.042		**.534**
11. B_PS_	0.79	2.53	0.68	2.87	0.53	−4.81	<.001	0.56	**.183**	**−.339**	**−.211**	**.291**	**−.454**	**.353**	.110	**−.171**	−.031	**.674**	

*^a^Values above the diagonal indicate coefficients for Japanese sample, and values below the diagonal indicates coefficients for Western sample. The coefficients shown in bold face were significant at p < .05. d, Cohen’s d statistics; α, Cronbach’s alpha coefficients; CAB, cognitive ability score described by unweighted mean POMP scores (0–100) of syllogism and subjective numeracy; RAT, rationality (five-point); EXP, experientiality (five-point); CRT, Cognitive Reflection Test; Cause, Causality scale of AHS (seven-point); Contra, Attitude toward Contradiction scale of AHS; Change, Perception of Change scale of AHS; Attention, Locus of Attention of AHS; B_PA_, belief in the paranormal (five-point); B_PS_, belief in pseudoscience (five-point). Of the above indicators, RAT and CRT are indices of Type-2 analytic thinking, whereas EXP is an index of intuitive thinking. All AHS subscales are indices of a holistic mode of thought.*

#### Cultural Differences and Determinants of Epistemically Suspect Beliefs

In order to identify potential determinants of ESBs we conducted multiple regression analyses predicting ESBs (i.e., paranormal beliefs and pseudoscientific beliefs; see Table 2). In order to identify whether effects of our predictors differed across cultures (i.e., were moderated by cultural affiliation), we adopted a hierarchical regression approach. We excluded cognitive ability and three subscales of the AHS (attitude toward contradiction, perception of change, and locus of attention) from these analyses because preliminary analyses failed to show any contribution of these variables to differences in ESBs. In the first step, all predictors were entered simultaneously into the model. Next, the interaction terms of sample and other predictors (i.e., rationality, experientiality, CRT, and causality) were entered. For paranormal beliefs, Δ*R*^2^s demonstrated a significant improvement in the prediction of paranormal beliefs by introducing interaction terms of sample and other predictors (Δ*R*^2^ = .02, *p* = .005). We also found significant improvements in predicting pseudoscientific beliefs (Δ*R*^2^ = .01, *p* = .043). The final models for both paranormal and pseudoscientific beliefs can be viewed in Table 2 (left panel).

**TABLE 2:** Regression analyses predicting beliefs by cognitive traits, cultural orientation, and interactions with sample.

	Study 1								Study 2							
	B_PA_ (adj. *R*^2^ = .39)				B_PS_ (adj. *R*^2^ = .33)				B_PA_ (adj. *R*^2^ = .22)				B_PS_ (adj. *R*^2^ = .30)			
Predictors	*b*	*SE*	β	*p*	*b*	*SE*	β	*p*	*b*	*SE*	β	*p*	*b*	*SE*	β	*p*
(Intercept)	2.33	0.17		<.001	2.29	0.12		<.001	2.24	0.18		<.001	2.26	0.12		<.001
Sample ^a^	0.57	0.10	.576	<.001	0.22	0.07	.343	.001	0.43	0.13	.474	.001	0.20	0.08	.318	.015
Gender ^b^	–0.45	0.10	–.454	<.001	–0.15	0.07	–.232	.027	–0.20	0.10	–.217	.041	–0.05	0.06	–.076	.450
Age	0.01	0.00	.058	.218	0.01	0.00	.187	<.001	0.00	0.01	.048	.398	0.01	0.00	.132	.015
RAT	0.00	0.01	.020	.790	–0.00	0.01	–.025	.758	–0.04	0.02	–.170	.041	–0.04	0.01	–.277	<.001
EXP	0.04	0.01	.183	.004	0.01	0.01	.077	.250	0.10	0.02	.431	<.001	0.06	0.01	.348	<.001
CRT	–0.31	0.06	–.358	<.001	–0.21	0.04	–.384	<.001	–0.21	0.06	–.271	.001	–0.21	0.04	–.376	<.001
Causality	0.04	0.02	.136	.023	0.04	0.01	.238	<.001								
DSS									0.29	0.12	.188	.016	0.02	0.08	.018	.804
Sample × RAT	0.01	0.02	.052	.609	0.00	0.01	.036	.738	0.08	0.03	.350	.003	0.04	0.02	.264	.019
Sample × EXP	0.05	0.02	.242	.016	0.03	0.01	.214	.042	–0.06	0.03	–.238	.036	–0.01	0.02	–.057	.596
Sample × CRT	0.29	0.09	.331	.001	0.15	0.06	.264	.013	0.16	0.09	.208	.057	0.07	0.06	.132	.203
Sample × Causality	–0.03	0.03	–.102	.290	–0.02	0.02	–.128	.206								
Sample × DSS									–0.14	0.19	–.089	.473	0.24	0.13	.217	.065

*All continuous variables except for age were mean-centered. ^a^1 = Japanese, 0 = Western. ^b^1 = men, 0 = women.*

We observed a significant gender difference for both ESBs in which women were more likely to have stronger ESBs compared to men (B_PA_: β = −.45, *p* < .001, B_PS_: β = −.23, *p* = .027). As predicted, CRT performance was negatively associated with ESBs (B_PA_: β = −.36, *p* < .001, B_PS_: β = −.38, *p* < .001), whereas a holistic understanding of causality was positively associated with ESBs (B_PA_: β = .14, *p* = .023, B_PS_: β = .24, *p* < .001). Additionally, we observed significant experientiality × sample (B_PA_: β = .24, *p* = .016, B_PS_: β = .21, *p* = .042) and CRT × sample interactions for both ESBs (B_PA_: β = .33, *p* = .001, B_PS_: β = .26, *p* = .013). Subsequent simple slope analyses revealed cultural affiliation to be a significant moderator of the association between experientiality and ESBs. The unstandardized simple slopes for Japanese participants were, B_PA_ = 0.08, *p* < .001, and B_PS_ = 0.04, *p* < .001, and B_PA_ = 0.04, *p* = .004, and B_PS_ = 0.01, *p* = .250 for Western participants. Lastly, we found cultural affiliation to be a significant moderator of the association between CRT performance and ESBs. The unstandardized simple slopes for Japanese participants were, B_PA_ = −0.02, *p* = .709, and B_PS_ = −0.07, *p* = .121, and B_PA_ = −0.31, *p* < .001, and B_PS_ = −0.21, *p* < .001 for Western participants. Therefore, CRT performance predicted ESBs in our Western sample but not Japanese sample, possibly reflecting differences in cultural values with regards to avoiding ESBs. On the other hand, a propensity to value intuition was a strong predictor of ESBs among Japanese participants, whereas it was not predictive for Western participants, particularly for pseudoscientific beliefs. Furthermore, the fact that a propensity toward multiple causality positively predicted pseudoscientific beliefs provides some support for the idea that holistic modes of thought can lead to the endorsement of ESBs. Of note however, the influence of thinking style along the analytic-holistic dimension did not vary across cultures.

## Study 2

Study 1 demonstrated that holistic understanding of causality partially explained participants’ endorsement of ESBs. However, participants’ attitude toward contradiction was not associated with endorsement of ESBs. This may be due to the fact that individuals’ attitude toward contradiction in the current context doesn’t reflect their propensity toward naïve dialecticism (i.e., the belief that things are changing continuously, tolerance of contradiction, and the preference for endorsing moderate options centered between two opposing options; cf. Zhang et al., 2015). Therefore, in Study 2, we further explored the association between dialectic thinking and endorsement of ESBs.

### Method

#### Participants

A sample of 316 participants were recruited from two online crowdsourcing platforms, 167 Japanese participants (56% female; *M*_age_ = 39.26, *SD*_age_ = 9.45) from CW and 149 Western participants (42% female; *M*_age_ = 30.60, *SD*_age_ = 10.04) from ProA. All participants received compensation (CW = 240 JPY; ProA = £2.00) upon completion of an approximately 16-min online questionnaire. As gender was a predictor in our regression analyses, two participants from ProA, and one from CW were excluded due to not reporting gender.

#### Materials

The materials used in Study 2 were nearly identical to those used in Study 1, with the following exceptions. First, we reduced the number of REI items we administered from 12 to 10 (now including five rational subscale and five experiential subscale items). These items were adopted from the REI-10 (Epstein et al., 1996), and assessed individual differences in thinking style. Second, we no longer administered the AHS, instead presenting participants with 32 Dialectical Self Scale items as our measure of holistic cognition (DSS; Spencer-Rodgers et al., 2004; the Japanese version of DSS items were adopted from Zhang et al., 2015). This scale measured participants’ degree of dialectical thinking (i.e., the degree to which participants are able to synthesize competing ideas or viewpoints) within the domain of self-perception (e.g., “When I hear two sides of an argument, I often agree with both”). Participants responded to each DSS item using a seven-point scale ranging from 1 (*Strongly disagree*) to 7 (*Strongly agree*). Responses to all 32 items were averaged for each participant to obtain a DSS score.

#### Procedure

Participants were administered an online questionnaire in which they were asked to respond to various items in the following order: pseudoscientific belief items, paranormal belief items, REI items, a syllogistic reasoning task, the SNS, the DSS, and the CRT. Following the completion of these tasks, participants were asked a series of demographic questions (i.e., age, gender, ethnicity, and native language). As in Study 1, all materials were presented in Japanese for CW participants and in English for ProA participants.

### Results and Discussion

Table 3 displays the descriptive statistics and bivariate correlations of all key variables. A series of independent samples *t*-tests demonstrated that our two samples did not differ in cognitive ability or CRT performance (both *p*s > .19). In contrast, we observed differences in our measures of paranormal beliefs, pseudoscientific beliefs, dialectical thinking, rationality, and experientiality between Japanese and Western samples. As in Study 1, Japanese participants endorsed more paranormal, *t*(313) = 4.42, *p* < .001, *d* = 0.50, and pseudoscientific beliefs, *t*(314) = 3.97, *p* < .001, *d* = 0.45. Japanese participants also demonstrated a greater degree of dialectic thinking compared to Western participants, *t*(314) = 9.75, *p* < .001, *d* = 1.10. Lastly, Western participants scored higher on both the rationality, *t*(314) = 6.50, *p* < .001, *d* = 0.73, and experientiality, *t*(314) = 6.40, *p* < .001, *d* = 0.72, subscales of the REI-10.

**TABLE 3:** Descriptive statistics and correlations of key variables (Study 2).

		West (*n* = 149)		JP (*n* = 167)		Sample difference			Pearson correlation coefficients ^a^							
	α	*M*	*SD*	*M*	*SD*	*t*	*p*	*d*	Age	CAB	RAT	EXP	CRT	DSS	B_PA_	B_PS_
Age		30.60	10.03	39.47	9.34	–7.92	<.001	0.92		.063	.118	−.098	**.163**	−.134	−.098	−.059
CAB		62.49	12.97	60.65	12.39	1.29	.199	0.15	.067		**.395**	**−.208**	**.441**	−.025	−.037	**−.304**
RAT	0.79	3.66	0.77	3.10	0.75	6.50	<.001	0.73	.105	**.368**		−.091	**.381**	**−.209**	.117	**−.171**
EXP	0.80	3.59	0.70	3.08	0.73	6.40	<.001	0.72	.127	.017	.092		**−.201**	**−.211**	**.217**	**.336**
CRT		1.41	1.15	1.39	1.19	0.10	.917	0.01	.054	**.588**	**.236**	−.015		−.057	-.064	**−.329**
DSS	0.83	3.62	0.59	4.20	0.45	–9.75	<.001	1.10	**−.171**	−.075	**−.252**	**−.166**	−.156		.019	.124
B_PA_	0.93	2.24	0.94	2.69	0.85	–4.42	<.001	0.50	.159	**−.275**	**−.221**	**.365**	**−.334**	**.197**		**.468**
B_PS_	0.79	2.48	0.69	2.76	0.57	–3.97	<.001	0.45	**.202**	**−.317**	**−.281**	**.312**	**−.390**	.070	**.624**	

*^a^Values above the diagonal indicate coefficients for Japanese sample, and values below the diagonal indicate coefficients for Western sample. The coefficients shown in bold face were significant at p < .05. All scales except DSS (seven-point) are five-point scales. As in Study 1, RAT and CRT are indices of Type-2 analytic styles of thinking and EXP is an index of intuitive thinking. DSS is an index of holistic thinking. DSS, Dialectical Self Scale; B_PA_, belief in the paranormal; B_PS_, belief in pseudoscience.*

#### Cultural Differences and Determinants of Epistemically Suspect Beliefs

As in Study 1, we conducted multiple regression analyses predicting ESBs (i.e., paranormal beliefs and pseudoscientific beliefs) in order to identify potential determinants of ESBs (see Table 2). We adopted a hierarchical regression approach to identify whether the effects of predictors were moderated by cultural affiliation. Consistent with Study 1, we found that women were more likely to endorse paranormal beliefs (β = −.32, *p* = .015) and older individuals more likely to endorse pseudoscientific beliefs (β = −.22, *p* = .041). Additionally, experientiality was positively associated with ESBs (B_PA_: β = .43, B_PS_: β = .35, *p*s < .001), while rationality (B_PA_: β = −.17, *p* = .041, B_PS_: β = −.28, *p* < .001) and CRT performance (B_PA_: β = −.27, *p* = .001, B_PS_: β = −.38, *p* < .001) was negatively associated with ESBs. Lastly, DSS scores were found to be positively associated with the endorsement of paranormal but not pseudoscientific beliefs (B_PA_: β = .19, *p* = .016, B_PS_: β = .02, *p* = .804).

We observed multiple interactions between various cognitive traits and cultural affiliation. For belief in the paranormal, we found significant rationality × sample (β = .35, *p* = .003) and experientiality × sample interactions (β = −.24, *p* = .036), as well as a marginally significant CRT × sample interaction (β = .21, *p* = .057). Deconstructing the rationality × sample interaction, we found a negative slope within our Western sample (unstandardized slope = −0.038, *p* = .040) and a positive slope within our Japanese sample (0.045, *p* = .015). Similarly, CRT performance was shown to be negatively associated with paranormal beliefs in our Western sample (−0.212, *p* < .001) and not associated with such beliefs in our Japanese sample (−0.058, *p* = .321). Deconstructing the experientiality × sample interaction, we found a positive simple slope in our Western sample (0.104, *p* < .001) and a more moderate, but significant, positive slope in our Japanese sample (0.050, *p* = .007). For belief in pseudoscience, we observed significant rationality × sample (β = .26, *p* = .019) and marginally significant DSS × sample interactions (β = .22, *p* = .065). Simple slope analyses revealed that rationality was negatively associated with belief for Western participants (−0.044, *p* < .001), but not associated with belief for Japanese participants (−0.001, *p* = .917). Thus, highly rational Westerners were less likely to endorse ESBs whereas rationality scores were not associated with ESBs in our Japanese sample. Conversely, DSS scores were positively associated with pseudoscientific belief in our Japanese sample (0.236, *p* = .015), but not in our Western sample (0.019, *p* = .814), demonstrating that Japanese participants high in dialectical thinking endorsed more pseudoscientific beliefs while the same was not true in our Western sample. Overall, the results of Study 2 provide further evidence suggesting that the mechanisms underlying ESBs differ across cultures. Specifically, rationality and Type-2 analytic thinking, were negatively correlated with ESBs in Westerners, but not Japanese participants. Furthermore, in some cases, these relations were reversed (i.e., positive) within our Japanese sample.

## Study 3

In Studies 1 and 2, we observed that a tendency toward holistic cognition was positively associated with ESBs. However, we failed to find an association between propensity toward contradiction and ESBs. As discussed earlier, the self-rated measure of attitude toward contradiction, namely, the attitude toward contradiction subscale of the AHS, may not reflect an individual’s dialectical thinking behavior. Therefore, Study 3 was conducted to see whether individuals who think dialectically have stronger paranormal and pseudoscientific beliefs than those who do not. To this end, participants in Study 3 were asked to indicate to what extent they endorse statements expressing epistemically suspect beliefs (pro-belief), as well as statements which explicitly deny epistemically suspect beliefs (anti-belief).

### Method

#### Participants

A sample of 301 participants were recruited from two online sources, 151 Japanese participants (69% female; *M*_age_ = 36.62, *SD*_age_ = 8.99) from CW and 150 Western participants (43% female; *M*_age_ = 32.91, *SD*_age_ = 11.42; 17% United States residents, 41% United Kingdom residents, 39% other) from ProA. All participants received compensation (CW = 240 JPY; ProA = £2.00) upon completion of an approximately 18-min online questionnaire. One participant from the CW sample was excluded from all analyses due to providing incomplete responses to belief tasks.

#### Materials

The materials used in Study 3 were similar to those used in Study 1, with the following exceptions. First, we administered only 12 AHS items (those from the causality and attitude toward contradiction subscales), removing the 12 AHS items associated with the perception of change and locus of attention subscales on account of responses to these items failing to explain endorsement of ESBs. However, we decided not to exclude six items from attitude toward contradiction subscale to see if it correlates with actual dialectical thinking behavior. Second, we chose five paranormal belief and five pseudoscientific belief items from Studies 1 and 2 (e.g., “Some people can have a dream that has predicted some future events”) and created anti-belief statements for each item (e.g., “No one can have a dream that has predicted future events”). The resulting 10 pairs of statements were divided into two sets, such that each set contained five pro- and five anti-belief items. We ensured that pro- and anti-belief statements for the same item were not included in the same set. As in Studies 1 and 2, participants rated their agreement with each presented statement on a seven-point scale ranging from “1 (*Strongly disagree*)” to “7 (*Strongly agree*).” A paranormal (B_PA_) and pseudoscience (B_PS_) score was calculated for each participant by taking the average of their responses to paranormal and pseudoscientific pro-belief statements, respectively.

Lastly, we assessed Dialectic Thinking (DT) using a methodology featured in past work (Zhang et al., 2015). In order to calculate a DT score for each participant, believability ratings for paired pro- and anti-belief statements were standardized (*Z*-scores) for each issue such that the midpoint “4” was set to equal zero. Next, both *Z*-scores were summed to create a DT score for a particular item. Finally, all five DT scores were averaged for paranormal (DT_PA_) and pseudoscientific items (DT_PS_), separately, creating a DT score pertaining to paranormal beliefs and another score pertaining to pseudoscientific beliefs.

*Z*-score P*_k_* (*Z*_*Pk*_) = (raw rating of Pro-belief statement of item *k −* 4)/SD_*P*_*_*k*_*_+*A*_*_*k*_*

*Z*-score A*_*k*_* (*Z*_*Ak*_) = (raw rating of Anti-belief statement of item *k −* 4)/SD_*P*_*_*k*_*_+*A*_*_*k*_*

$$D\,T\,s\,c\,o\,r\,e=\frac{1}{5}\,\sum_{k=1}^{5}\left|Z_{P\,k}+Z_{A\,k}\right|$$

#### Procedure

Participants were administered an online questionnaire in which they were asked to respond to various items in the following order: a belief task, REI-10 items, the AHS, a second belief task, a syllogistic reasoning task, the SNS and the CRT. Following the completion of these tasks, participants were asked a series of demographic questions (i.e., age, gender, nationality, ethnicity, and native language). As in Studies 1 and 2, all materials were presented in Japanese for CW participants and in English for ProA participants.

### Results and Discussion

Table 4 shows the descriptive statistics and bivariate correlations for all key variables. Once again, we conducted a series of independent samples *t*-tests to investigate differences across our Japanese and Western samples. We observed no differences between Western and Japanese participants with regards to their attitude toward contradiction, CRT performance, cognitive ability, or endorsement of pseudoscientific beliefs (all *p*s > .09). Replicating the findings of Studies 1 and 2, Western participants scored higher on our measure of rationality, *t*(299) = 6.14, *p* < .001, *d* = 0.72, and endorsed less paranormal beliefs, *t*(299) = 5.15, *p* < .001, *d* = 0.60. Western participants also scored higher on our measure of experientiality, *t*(299) = 5.29, *p* < .001, *d* = 0.61, whereas Japanese participants scored higher in causal perception (as measured by the causality subscale of the AHS), *t*(299) = 3.50, *p* < .001, *d* = 0.40. Interestingly, DT scores were not correlated with either causality or contradiction scores.

**TABLE 4:** Descriptive statistics and correlations of key variables (Study 3).

	West (*n* = 135)		JP (*n* = 126)		Sample difference			Pearson correlation coefficients ^a^										
	*M*	*SD*	*M*	*SD*	*t*	*p*	*d*	1.	2.	3.	4.	5.	6.	7.	8.	9.	10.	11.
1. Age	32.91	11.42	36.62	8.99	−3.13	.002	0.36		.100	.124	−.032	.127	.077	.089	.026	**.264**	−.158	**−.234**
2. CAB	61.33	19.07	57.79	17.15	1.69	.092	0.20	−.079		.117	−.046	**.454**	.068	−.073	−.104	.022	−.053	−.089
3. RAT	3.69	0.71	3.15	0.79	6.18	<.001	0.72	−.118	**.210**		.026	.129	**.208**	−.120	.078	.099	−.063	−.037
4. EXP	3.65	0.72	3.32	0.67	5.29	<.001	0.61	.029	**−.251**	−.057		−.055	.114	.143	**.226**	.146	.038	.007
5. CRT	1.31	1.16	1.43	1.00	−0.99	.322	0.11	**−.189**	**.376**	**.182**	−.145		−.057	.062	**−.305**	−.096	−.143	−.059
6. Cause	5.07	0.88	5.43	0.90	−3.50	<.001	0.40	−.087	−.128	.079	**.167**	.075		**.178**	**.262**	**.196**	.046	.008
7. Contra	4.77	0.98	4.77	0.82	0.03	.978	0.00	−.053	−.111	**−.222**	−.024	**−.179**	.158		−.010	.053	−.040	.082
8. B_PA_	3.03	1.33	3.83	1.38	−5.15	<.001	0.60	.058	**−.223**	−.107	**.385**	**−.225**	**.417**	.070		**.430**	−.067	−.159
9. B_PS_	3.54	0.87	3.63	0.76	1.20	.229	0.14	**.255**	**−.291**	**−.174**	**.283**	**−.338**	**.272**	**.161**	**.513**		−.059	**−.366**
10. DT_PA_	0.36	0.33	0.38	0.27	−0.48	.630	0.06	.042	−.137	−.139	**.224**	−.048	.034	−.004	.085	**.196**		**.164**
11. DT_PS_	0.54	0.38	0.63	0.42	−2.00	.046	0.23	**−.162**	−.156	.007	.107	−.109	−.072	.087	−.087	−.046	**.226**	

*^a^Values above the diagonal indicate coefficients for Japanese sample, and values below the diagonal indicates coefficients for Western sample.*

*The coefficients shown in bold face were significant at p < .05. All scales except subscales of AHS (Cause and Contra; seven-point) are five-point scales. DT_PA_, Dialectic Thinking score for paranormal items; DT_PS_, Dialectic Thinking score for pseudoscience items.*

#### Cultural Differences and Determinants of Epistemically Suspect Beliefs

As in Studies 1 and 2, we investigated potential determinants of ESBs by conducting hierarchical regression analyses. We excluded measures of cognitive ability and attitude toward contradiction as preliminary analyses failed to show any contribution of these variables in predicting the endorsement of ESBs. With the exception of these exclusions, all predictors were entered simultaneously into our model in Step 1. In Step 2, we entered the interaction terms with sample. The introduction of these interaction terms marginally improved our model for pseudoscientific beliefs (Δ*R*^2^ = .02, *p* = .056), but not paranormal beliefs (Δ*R*^2^ = .01, *p* = .143). In Step 3, we entered DT scores for both ESB domains (pseudoscientific and paranormal beliefs) as well as the DT × sample interaction into our model. These additions improved our model for pseudoscientific beliefs (Δ*R*^2^ = .04, *p* < .001), but not paranormal beliefs (Δ*R*^2^ = .002, *p* = .243). Both Step 2 (without DT scores) and Step 3 (with DT scores) models for both paranormal and pseudoscientific beliefs can be viewed in Table 5.

**TABLE 5:** Regression analyses predicting beliefs by cognitive traits, cultural orientation, dialectical thinking, and interactions with sample (Study3).

	B_PA_								B_PS_							
	Without DT				With DT				Without DT				With DT			
Predictors	*b*	*SE*	β	*p*	*b*	*SE*	β	*p*	*b*	*SE*	β	*p*	*b*	*SE*	β	*p*
(Intercept)	3.09	0.27		<.001	3.12	0.28		<.001	3.29	0.16		<.001	3.39	0.19		<.001
Sample ^a^	0.77	0.16	.548	<.001	1.00	0.24	.552	<.001	–0.22	0.10	–.266	.031	0.12	0.15	–.206	.438
Gender ^b^	–0.35	0.15	–.247	.022	–0.35	0.15	–.248	.022	–0.19	0.09	–.236	.041	–0.15	0.09	–.184	.106
Age	0.01	0.01	.036	.476	0.00	0.01	.028	.574	0.02	0.00	.232	<.001	0.02	0.00	.199	<.001
RAT	–0.03	0.03	–.077	.328	–0.03	0.03	–.077	.330	–0.03	0.02	–.123	.142	–0.03	0.02	–.127	.120
EXP	0.10	0.03	.248	.001	0.10	0.03	.248	.001	0.04	0.02	.175	.023	0.04	0.02	.185	.015
CRT	–0.19	0.09	–.144	.031	–0.19	0.09	–.146	.030	–0.19	0.05	–.249	.001	–0.20	0.05	–.263	<.001
Causality	0.10	0.02	.379	<.001	0.10	0.02	.379	<.001	0.05	0.01	.320	<.001	0.05	0.01	.316	<.001
DT ^c^					0.01	0.30	.002	.970					–0.07	0.16	–.035	.649
RAT × sample	0.05	0.04	.137	.197	0.05	0.04	.131	.218	0.03	0.02	.169	.136	0.03	0.02	.167	.132
EXP × sample	–0.02	0.04	–.044	.671	–0.02	0.04	–.040	.701	–0.01	0.03	–.039	.722	–0.01	0.02	–.051	.639
CRT × sample	–0.17	0.13	–.131	.193	–0.19	0.13	–.146	.149	0.13	0.08	.169	.117	0.12	0.08	.161	.126
Causality × sample	–0.05	0.03	–.206	.044	–0.05	0.03	–.199	.051	–0.03	0.02	–.213	.050	–0.03	0.02	–.198	.064
DT × sample ^c^					–0.61	0.47	–.130	.194					–0.49	0.21	–.241	.019
Adjusted *R*^2^	.31				.31				.21				.25			

*All continuous variables except for age were mean-centered.*

*^a^1 = Japanese, 0 = Western. ^b^1 = men, 0 = women. ^c^DT, Dialectical Thinking score of each domain (DT_PA_ for B_PA_ and DT_PS_ for B_PS_.*

As in Study 1, our measure of experientiality was positively associated with ESBs (β_PA_ = .25, β_PS_ = .19, *p*s < .05) and CRT performance was negatively associated with ESBs (β_PA_ = −.14, β_PS_ = −.26, *p*s < .05). Additionally, holistic understanding of causality was positively associated with ESBs (β_PA_ = .38, β_PS_ = .32, *p*s < .001). Therefore, consistent with past theorizing, the tendency to think holistically may leave one susceptible to ESBs while analytic thinking may protect against such beliefs. Unlike in Studies 1 and 2, significant interactions with sample were found only for dialectic thinking pertaining to pseudoscientific beliefs (β = −.24, *p* = .019). Simple slope analysis revealed that dialectic thinking was positively associated with pseudoscientific beliefs in our Western sample (0.047, *p* < .001), but not our Japanese sample (0.018, *p* = .116). Thus, Western participants high in dialectical thinking endorsed more pseudoscientific beliefs while the same was not true in our Japanese sample. Interestingly, the actual behavior of dialectic thinking exhibited a slightly different pattern of results from self-report measures of dialecticism. Overall, Study 3 provided further evidence suggesting that the mechanisms underlying ESBs differ across cultures.

## Study 4

Study 4 explored cultural differences in receptivity to pseudo-profound bullshit. The fact that individuals’ receptivity to pseudo-profound bullshit is positively associated with their acceptance of ESBs suggests common cognitive mechanisms underlying bullshit receptivity and acceptance of ESBs. While past work has examined the mechanisms underlying bullshit receptivity (Pennycook et al., 2015a; Walker et al., 2019), no work (to our knowledge) has examined these mechanisms cross-culturally. As evidenced by Studies 1–3, the association between various individual difference measures (e.g., analytical thinking) and ESBs (e.g., pseudoscientific belief) may vary between cultures. Similar to ESBs, bullshit receptivity has been found to be negatively associated with analytical thinking (Pennycook et al., 2015a; Pennycook and Rand, 2020), however, such studies have relied heavily on WEIRD samples. Thus, in Study 4 we aim to investigate whether the same mechanisms (e.g., analytical thinking) underlie bullshit receptivity across Western (American) and Eastern (Japanese) participants. Furthermore, recent studies have demonstrated how individual differences in illusory pattern perception relate to bullshit receptivity (Walker et al., 2019) and various ESBs (van Prooijen et al., 2018). Therefore, in Study 4, we also assessed participants’ performance on a pattern perception task. We predict that individuals demonstrating greater illusory pattern perception will also show a greater receptivity to pseudo-profound statements. Additionally, we assess whether the association between illusory pattern perception and bullshit receptivity differs between American and Japanese individuals.

### Method

#### Participants

A sample of 401 participants were recruited from two online crowdsourcing platforms, 201 Japanese participants (46% female; *M*_age_ = 39.45, *SD*_age_ = 10.02) from CW and 200 U. S. residents (37% female; *M*_age_ = 34.30, *SD*_age_ = 10.67) from Amazon’s Mechanical Turk (MTurk). All participants received compensation (CW = 270 JPY; Mturk = $2.50 USD) upon completion of an approximately 15-min online questionnaire.

#### Materials

In Study 4, we administered the CRT and AHS (24-item version; Choi et al., 2007) along with two new measures: a modified snowy pictures task (Whitson and Galinsky, 2008) and a profundity judgment task (Pennycook et al., 2015a).

##### Modified Snowy Pictures Task

The modified snowy pictures task (MSPT; Whitson and Galinsky, 2008) was used to measure participants’ ability to detect real patterns and avoid endorsing illusory patterns. Participants were presented with 24 pictures, 12 of which contained a difficult to perceive object and 12 of which contained only visual noise. For each picture, they were asked whether the presented image contained an object and responded with either a “yes” or “no” response. A non-illusory pattern perception score was calculated for each participant by determining how many object-present items they correctly endorsed as containing an object. Similarly, an illusory pattern perception score was calculated for each participant by determining how many object-absent items participants correctly endorsed as not containing an object. Thus, higher scores for object-present and object-absent items were indicative of a greater tendency to identify real patterns and a reduced tendency to endorse illusory patterns, respectively.

##### Profundity Judgments

Participants were presented with 30 statements (10 pseudo-profound bullshit statements, 10 motivational quotations, and 10 mundane statements) and were asked to assess the profundity of each statement on a five-point scale ranging from “1 = *Not at all profound*” to 5 “*Very profound*.” All statements originated from Pennycook et al. (2015a)^6^. Pseudo-profound bullshit statements were originally obtained from two websites^7^ able to create superficially impressive yet meaningless statements by randomly arranging a list of profound sounding words in a way that maintains syntactic structure. A bullshit receptivity score (BSR) was created for each participant by calculating their mean profundity rating given to pseudo-profound bullshit items (e.g., “Hidden meaning transforms unparalleled abstract beauty”), with higher scores indicating greater bullshit receptivity. Ten motivational quotations and mundane statements were also presented to participants, contrasting the meaningless nature of pseudo-profound bullshit statements. All motivational quotations were originally obtained via an internet search and were designed to communicate something meaningful and reasonably profound (e.g., “A wet man does not fear the rain”). Mundane statements also communicated something meaningful, however, they were designed so that the message being communicated was banal (e.g., “New born babies require constant attention”). Identical to that of the BSR, a motivational quotation (MQ) and mundane statement (MS) score was calculated for each participant by determining their mean profundity rating given to motivational and mundane items, respectively.

#### Procedure

Participants were once again administered an online questionnaire for which they completed 30 profundity judgments, 24 MSPT items, 24 AHS items, the CRT, and a host of demographic questions in that order. As in previous studies, all materials were presented in Japanese for CW participants and in English for Mturk participants.

### Results and Discussion

Descriptive statistics and bivariate correlations for all variables can be viewed in Table 6. Independent samples *t*-tests revealed that Japanese participants scored higher on 3 of the 4 sub-scales of the AHS [Causality: *t*(399) = 2.17, *p* = .030, *d* = 0.22; Change: *t*(399) = 3.83, *p* = .001, *d* = 0.38; Attention: *t*(399) = 2.66, *p* = .008, *d* = 0.27] demonstrating an overall greater tendency to engage in holistic thinking compared to United States participants. No differences were observed between Japanese and United States participants with regards to CRT performance, illusory pattern perception or bullshit receptivity (all *p*s > .220). Furthermore, consistent with past work (Walker et al., 2019), bullshit receptivity was found to positively correlate with illusory pattern perception [United States: *r*(198) = .33, *p* < .001; Japan: *r*(198) = .25, *p* < .001]. Also consistent with past work (Pennycook et al., 2015a; Pennycook and Rand, 2020), CRT performance was found to negatively relate to bullshit receptivity within our United States sample, *r*(198) = −.38, *p* < .001. Interestingly, no association was found between CRT performance and bullshit receptivity for Japanese participants, *r*(198) = −.05, *p* = .487.

**TABLE 6:** Descriptive statistics of cognitive traits and beliefs (Study 4).

	West (*n* = 181)		JP (*n* = 177)		Sample difference			Pearson correlation coefficients ^a^										
	*M*	*SD*	*M*	*SD*	*t*	*p*	*d*	1.	2.	3.	4.	5.	6.	7.	8.	9.	10.	11.
1. Age	34.30	10.67	39.45	10.02	−4.97	<.001	0.50		**.165**	.046	.130	.079	−.040	.075	**.157**	−.101	−.021	−.056
2. CRT	1.54	0.70	1.43	1.11	1.21	.227	0.12	.057		−.087	.084	.050	−.127	**.151**	−.087	−.049	**−.160**	**−.400**
3. Pattern (P)	9.03	2.60	8.55	2.37	1.92	.055	0.19	.084	.059		**−.523**	**.160**	−.013	−.012	.139	**.155**	**.234**	.036
4. Patten (A)	9.89	2.33	9.61	2.44	1.21	.227	0.12	.014	**.165**	**−.376**		−.054	.008	**.146**	−.071	**−.250**	**−.213**	**−.242**
5. Cause	5.18	0.92	5.36	0.73	−2.17	.030	0.22	−.035	.020	.064	.069		.139	**.142**	**.341**	.103	**.207**	−.026
6. Contra	4.91	1.05	4.73	0.73	2.00	.046	0.20	−.116	.066	.108	−.050	**.237**		−.133	**.292**	.024	**.177**	.079
7. Change	4.53	0.97	4.86	0.75	−3.83	<.001	0.38	.084	**.142**	.097	**.225**	.014	−.003		−.111	−.023	−.002	**−.152**
8. Attention	4.95	0.94	5.16	0.61	−2.66	.008	0.27	−.009	**−.159**	.010	−.004	**.230**	**.244**	**−.146**		.087	**.167**	−.006
9. BSR	2.60	1.00	2.58	0.75	0.28	.778	0.03	**−.157**	**−.382**	.116	**−.334**	**.147**	.104	**−.169**	.089		**.527**	**.332**
10. MQ	3.20	0.76	2.99	0.65	2.94	.003	0.29	−.033	**−.178**	.089	**−.270**	.130	**.267**	−.123	**.193**	**.575**		**.442**
11. MS	1.50	0.86	1.96	0.83	−5.52	<.001	0.55	−.112	**−.358**	−.096	**−.453**	−.062	−.027	**−.516**	.100	**.452**	**.292**	

*Pattern (P), number of correct responses to 12 object-present items where an object was embedded; Pattern (A), number of correct responses to 12 object-absent items where an object was not embedded (lower scores illustrate greater illusory pattern perception); BSR, mean profundity rating given to pseudo-profound bullshit statements; MQ, mean profundity rating given to motivational quotes; MS, mean profundity rating given to mundane statements. All profundity judgments are five-point scales and subscales of AHS are seven-point scales.*

*^a^Values above the diagonal indicate coefficients for Japanese sample, and values below the diagonal indicates coefficients for Western sample. The coefficients shown in bold face were significant at p < .05.*

Next, we conducted multiple regression analyses predicting bullshit receptivity with individual differences in CRT performance, holistic cognition (as measured by the AHS), illusory pattern perception, and interactions of these variables with culture. Since preliminary analyses revealed that the AHS subscales of attitude toward contradiction, perception of change, and locus of attention failed to predict individuals’ receptivity to bullshit, the causality subscale was the only measure of holistic thinking included in our model. Analyses of Δ*R*^2^ showed that our model was significantly improved at all steps (Step 1: Δ*R*^2^s = .144, *p* < .001; Step 2: Δ*R*^2^s = .057, *p* < .001). The final model can be viewed in Table 7. These results show that individual differences in CRT performance (β = −.50, *p* < .001), illusory pattern perception (β = −.34, *p* < .001), and holistic cognition (as measured by the causality subscale of the AHS; β = .18, *p* = .002) predicted bullshit receptivity. Furthermore, we observed a significant CRT × sample interaction (β = .48, *p* < .001). A simple slope analysis revealed that CRT was negatively associated with bullshit receptivity within our United States sample (−0.48, *p* < .001), and shared no association with bullshit receptivity in our Japanese sample (−0.023, *p* = .666), suggesting that the mechanisms underlying bullshit receptivity, like ESBs, may differ across cultures.

**TABLE 7:** Regression analysis predicting bullshit receptivity by reflective thinking, holistic cognition, illusory pattern perception, and interactions with sample (Study 4).

Predictors	*b*	*SE*	β	*p*
(Intercept)	2.91	0.16		<.001
Sample ^a^	–0.05	0.08	–.048	.595
Gender ^b^	0.10	0.08	.114	.230
Age	–0.01	0.00	–.109	.022
Pattern absent	–0.13	0.03	–.342	<.001
CRT	–0.48	0.08	–.503	<.001
Causality	0.03	0.01	.179	.002
Sample × Pattern	0.06	0.03	.152	.100
Sample × CRT	0.46	0.10	.479	<.001
Sample × Causality	–0.01	0.02	–.075	.425

*Pattern absent, number of correct responses to object-absent items where an object was not embedded (lower scores illustrate greater illusory pattern perception). Adjusted R^2^ = .182, F(9,390) = 10.90, p < .001.*

*^a^1 = Japanese, 0 = Western. ^b^1 = men, 0 = women.*

## General Discussion

The present study investigated cultural differences related to the endorsement of ESBs and pseudo-profound bullshit. Specifically, we examined the cognitive mechanisms supporting these beliefs across Eastern (Japanese) and Western (United States and Western Europe) cultures, revealing the generality versus specificity of cognitive mechanisms proposed in the literature surrounding ESBs. A number of key findings emerged from the current set of studies. First, consistent with past work (Shiah et al., 2010), Easterners, such as Japanese participants, showed stronger paranormal beliefs (Studies 1--3) and some evidence of stronger pseudoscientific beliefs (Studies 1 and 2) compared to Western participants^8^. Conversely, receptivity to pseudo-profound bullshit did not differ across cultures.

Individual differences in holistic cognition partly explained differences in ESBs and bullshit receptivity. Specifically, individual differences in the holistic understanding of causality (i.e., multiple and complex causality) played a key role in explaining differences in ESBs and bullshit receptivity. However, contrary to expectations, participants’ attitudes toward contradictions failed to explain ESBs and bullshit receptivity, suggesting that the stronger endorsement of ESBs in our Japanese sample was not due to a cultural difference in receptiveness to contradiction. Furthermore, individual differences in thinking style also explained differences in ESBs and bullshit receptivity, with a propensity to engage in analytic thinking negatively associated with the endorsement of ESBs and pseudo-profound bullshit statements as profound. Interestingly, the association between Type-2 analytic thinking (as measured by the CRT and rationality subscale of the REI) and ESBs differed across cultures. That is, for Westerners, individuals high in analytic thinking were less likely to endorse ESBs and pseudo-profound bullshit whereas no association was found between analytic thinking and either ESBs or bullshit receptivity in our Japanese sample. Thus, the present findings suggest that a propensity to engage in analytic thinking may protect against ESBs in Western individuals, but may not do so across cultures (specifically Eastern cultures).

Cultural psychologists have proposed a framework to understand cultural differences in cognitive styles between Western and Eastern individuals, distinguishing between analytic and holistic cognition (Nisbett et al., 2001; Nisbett and Miyamoto, 2005; Ishii, 2013). Related to this distinction, we hypothesized that individuals demonstrating a high tolerance for contradictions would be more likely to endorse ESBs, as we suspected that those accepting contradictions would tend to perceive pro-ESB statements as plausible even when they did not specifically endorse the ESB themselves. However, our results suggest that it is not a tendency to accept contradictions that positively relates to the endorsement of ESBs, but rather the consideration that causality is complex and multifaceted and that events may have multiple causes which need not necessarily contradict. Holistic thinkers, therefore, may be less impacted by conflicts caused by scientifically unsound claims and be more open to alternative explanations of events.

The culture (i.e., holistic cognition) hypothesis may be challenged by the fact that, with the exception of Study 3, our results failed to show significant sample by holistic cognition interactions. Nevertheless, it is possible that the present findings arose, at least in part, due to the mixture of falsifiable and unfalsifiable statements within ESB items. That is, the endorsement of an empirically falsifiable belief (e.g., a pseudoscientific belief) may be more strongly associated with an analytical mode of thought compared to unfalsifiable beliefs, on account of falsifiability being connected to the type of scientific, deductive, linear thinking that is characteristic of analytic thinking. Consistent with this claim, the observed negative association between CRT and ESBs was generally stronger when evaluating participants’ endorsement of falsifiable pseudoscientific beliefs compared to less readily falsifiable paranormal ones.

Additionally, within three of our four studies, we observed a sample by CRT interaction on ESBs and bullshit receptivity. Therefore, individuals’ CRT performance (an assessment of their tendency to engage in Type-2 analytic thinking) related differently to endorsement of ESBs and receptiveness to pseudo-profound bullshit across cultures. Consistent with past work (Pennycook et al., 2012, 2015a), CRT performance was negatively associated with ESBs and bullshit receptivity within our Western sample, suggesting that more deliberative modes of thinking may protect against these seemingly irrational beliefs. However, a different pattern of results was observed in our Japanese samples, for which CRT was unrelated to the endorsement of ESBs and pseudo-profound bullshit. Therefore, the present study suggests that the often observed negative association between Type-2 analytic thinking and various ESBs may be exclusive to Western individuals.

Of course, it remains an open question as to *why* Type-2 analytic thinking relates differently to the endorsement of ESBs across cultures. One possibility is that measures of analytic thinking (such as the CRT) in part measure a tendency to oppose cultural norms. In support of this claim is the finding that cognitive reflection is positively associated with religious disbelief within highly religious countries but shares no association with religious disbelief in less religious countries (Gervais et al., 2018). If true, we may expect the association between cognitive reflection and ESBs to differ between cultures with different cultural norms surrounding ESBs. Notably, in the present study, the effects of CRT on pseudoscientific beliefs were observed to be roughly the same across cultures, as opposed to paranormal beliefs which differed across cultures. One possible explanation for the different results observed for pseudoscientific and paranormal beliefs is that both Western and Eastern individuals may hold a similar norm against endorsing readily falsifiable pseudoscientific claims, while a norm against endorsing less readily falsifiable paranormal beliefs may be stronger among Western individuals. Therefore, in the present context, the weak association between cognitive reflection and ESBs (particularly paranormal beliefs) in our Japanese sample may arise due to the fact that these beliefs may not necessarily violate prevailing cultural norms, while the same may not be true of a Western sample.

## Summary

The present research investigates cultural differences in ESBs and bullshit receptivity. Critically, we demonstrate how the importance of Type-2 analytic thinking for suppressing ESBs (often observed in Western samples) fails to generalize across cultures (i.e., Japanese samples). Of course, the current work is not intended to be definitive; therefore, future studies should explore how factors such as analytic thinking predict other important real-world beliefs (e.g., endorsement of conspiracy theories and “fake news”) across cultures. Furthermore, the underlying mechanisms explaining what cultural factors elicit cultural differences regarding the endorsement of ESBs remains an open question. Nevertheless, we provide initial evidence demonstrating that not only does the endorsement of ESBs differ across cultures, but so too might the cognitive mechanisms supporting these beliefs, calling into question the generality of various cognitive mechanisms often thought to support ESBs.

## Data Availability Statement

Data and analysis scripts of all studies are available at https://osf.io/cbfwj/.

## Ethics Statement

The studies involving human participants were reviewed and approved by the Research Ethics Committee at Hokusei Gakuen University and the Office of Research Ethics, at the University of Waterloo. All participants provided their written informed consent to participate in this study.

## Author Contributions

YM designed the project, conducted empirical studies and statistical analyses, and drafted the manuscript. AW and MT co-designed and conducted Study 4 and revised the manuscript. JF supervised the project and critically reviewed the manuscript. All authors discussed the results and commented on the manuscript.

## Conflict of Interest

The authors declare that the research was conducted in the absence of any commercial or financial relationships that could be construed as a potential conflict of interest.

## Publisher’s Note

All claims expressed in this article are solely those of the authors and do not necessarily represent those of their affiliated organizations, or those of the publisher, the editors and the reviewers. Any product that may be evaluated in this article, or claim that may be made by its manufacturer, is not guaranteed or endorsed by the publisher.

## Footnotes

1 We did not conduct an *a priori* power analysis to determine sample size for this study. Instead, decisions regarding sample size were made with reference to related past work (e.g., Pennycook et al., 2012; Majima, 2015). We also decided to include Prolific participants having non-European origin into our Western sample since our preliminary analysis revealed that the overall results were not different when we excluded these participants. The number of corresponding participants were 6, 13, 4 and 51 in Studies 1 through 4, respectively. Similarly, CrowdWorks participants with a non-Asian origin were labeled as Japanese participants, since preliminary analysis also revealed that the results were not different when we excluded non-Asian CrowdWorks participants. The number of non-Asian CW participants were 0, 10, 1 and 24 in Studies 1 through 4, respectively. Some studies suggest that people of Asian ethnicity living in Western countries provide responses resembling an intermediate between Asian people living in Eastern countries and European people living in Western countries (Norenzayan et al., 2002). Therefore, it is noteworthy that the present results (featuring our full sample) did not differ when excluding Japanese participants with a non-Asian origin and Western participants with an Asian origin.

2 The Japanese translations for R-PBS items were drawn from two previous works (Nakajima et al., 1992, 1993).

3 Translations for BPS and AHS items were created as following. First, the first author translated items into Japanese. Next, another scholar working as an English teacher at the same university of the first author translated items back into English. The final translation was decided through discussion between the above two people.

4 Responses to nationality, ethnicity and native language questions were originally included as indicators to detect participants from a different culture than the one in which they were currently living. However, as noted in text footnote 1, we decided not to exclude participants on this basis. Therefore, we did not use these variables in the subsequent analysis.

5 Note that the reliability scores for AHS subscales were similar to those found in the original study (α ranged from .56 to .71; Choi et al., 2007).

6 The Japanese version of the profundity judgment task was created through the following procedure. First, the first author translated items into Japanese. Next, a professional English-proofing company translated the Japanese version of all items back into English and the accuracy of the translation was checked against the original text. Based on their suggestions, the first author made minor revisions to the translation, and finally, the first and second authors decided on the final translation by consensus.

7 http://wisdomofchopra.com and http://sebpearce.com/bullshit/

8 However, it is worth noting that participants’ endorsement of ESBs in the present research may have been affected by factors other than the believability of statements. For example, past work has found evidence of a midpoint preference among Japanese participants (Heine and Lehman, 1999). Thus, factors not related to participants’ endorsement of ESBs may have nevertheless contributed to these differences.
