# Supplementary material for: Culture as a Moderator of Epistemically Suspect Beliefs
Source: Front Psychol. 2022 Feb 9;13:745580. doi: 10.3389/fpsyg.2022.745580 (PMC8863589; doi:10.3389/fpsyg.2022.745580)
Supplement: Supplementary file 1 [file Data_Sheet_1.pdf]

Supplementary Material

Culture as a Moderator of Epistemically Suspect Beliefs

1 Study Materials

1.1 Study 1

Paranormal Belief Scale

Please answer the following questions by using a number between 1 and 5 to indicate your answer where:

1 = Extremely Unlikely, 2 = Unlikely, 3 = Unsure, 4 = Possible, 5 = Certain

1. Do you think that it is feasible for a person to have had a dream that has predicted some future event (a precognitive dream)? (P)
2. Is it conceivable for a person to be in communication with someone else using telepathy? (P)
3. How likely is it that you possess some form of 'psychic ability'? (P)
4. Do you believe in the existence of ESP (extrasensory perception)? (P)
5. Do you believe in the existence of PK (psychokinesis, i.e. the action of mind over matter)? (P)
6. Do you believe that ESP/PK can be demonstrated in the laboratory? (P)
7. How likely is it that your mind or soul can leave your body and travel (astral projection)? (S)
8. Do you believe that reincarnation does occur? (S)
9. Do you think that it is possible to communicate with the dead? (S)
10. Do you believe in the existence of black magic? (W)
11. Do you believe that witches do exist? (W)
12. Do you think that it is possible to cast spells on persons through the use of formulas and incantations? (W)

(P) items were drawn from Psi Questionnaire (Roberts & Seager, 1999), (S) were drawn from Spirituality subscale of Revised Paranormal Belief Scale (Tobacyk, 2004), and (W) were drawn from Witchcraft subscale from R-RPBS.

Belief in the pseudoscience

In the following pages, you can find one statement at a page. Please choose a number between 1 and 5 to indicate your opinion.

1: **Disagree** - 3: Neither disagree nor agree - 5: **Agree**

1. Homoeopathic remedies foster spontaneous healing. (M)
2. Excessive videogame playing can cause damage to the prefrontal cortex. (M)
3. An ionic detox bath that has an electric current passed through it draws out the toxins from our body. (M)
4. Negative ions in the air promote our physical and mental health. (M)
5. Soaking hands and feet in hot water infused with mineral germanium stimulates circulation, promotes recovery from fatigue and improves a stiff neck. (M)
6. Nigari (magnesium chloride extracted from seawater that used in the preparation of Japanese food, Tofu) has a beautifying and slimming effect. (M)
7. Childhood vaccines are one causal factor in the development of autism. (L)
8. Prayer is effective at treating people with terminal diseases. (L)
9. Reiki healing, otherwise known as Palm healing, is effective in healing the body. (L)
10. It has been scientifically proven that fatty acid supplements (omega-3 and omega-6, such as Docosahexaenoic acid and Linoleic acid) have a positive effect on academic achievement. (D)
11. If pupils do not drink sufficient amounts of water (=6–8 glasses a day) their brains shrink. (D)
12. Children are less attentive after consuming sugary drinks, and/or snacks. (D)

(M) items were drawn from Belief in the non-paranormal pseudoscience scale (Majima, 2015), (L) were drawn from Pseudoscience items of Belief survey (Lobato et al., 2014), and (D) were drawn from Neuromyths items (Dekker et al., 2012).

<u>Rational-Experiential Inventory (REI; Study 1, Naito et al., 2004; Pacini & Epstein, 1996)</u>

To what extent are the following statements true of yourself? Please answer by using a number between 1 and 5.

1: Definitely **<u>not true</u>** of myself - 3: Neither true nor false - 5: Definitely **<u>true</u>** of myself

1. I enjoy intellectual challenges. (RE)
2. I enjoy solving problems that require hard thinking. (RE)
3. I prefer complex problems to simple problems. (RE)
4. I am not very good at solving problems that require careful logical analysis. (RA) (R)
5. I am not a very analytical thinker. (RA) (R)
6. Reasoning things out carefully is not one of my strong point. (RA) (R)
7. I like to rely on my intuitive impressions. (EE)
8. I often go by my instincts when deciding on a course of action. (EE)
9. I don't think it is a good idea to rely on one's intuition for important decisions. (EE) (R)
10. Using my gut feelings usually works well for me in figuring out problems in my life. (EA)
11. I believe in trusting my hunches. (EA)
12. If I were to rely on my gut feelings, I would often make mistakes. (EA) (R)

RE = Rational Engagement, RA = Rational Ability, EE = Experiential Engagement, EA = Experiential Ability, (R) = reverse coded items

All rational items (engagement and ability) were averaged to obtain the Rationality score. Similarly, the Experientiality score was calculated as a mean of 6 experiential items.

Cognitive Reflection Test

See Frederick (2005) for details (we adopted standard 3-item version of CRT).

Analysis-Holism items (Study 1, Choi et al., 2007)

Please read the following statements and indicate your opinion by using a number between 1 and 7.

1: Strongly **Disagree** - 7: Strongly **Agree**

1. Everything in the universe is somehow related to each other. (CA)
2. Nothing is unrelated. (CA)
3. Everything in the world is intertwined in a causal relationship. (CA)
4. It is more desirable to take the middle ground than go to extremes. (CO)
5. When disagreement exists among people, they should search for ways to compromise and embrace everyone's opinions. (CO)
6. It is more important to find a point of compromise than to debate who is right/wrong, when one's opinions conflict with other's opinions. (CO)
7. Every phenomenon in the world moves in predictable directions. (CH)
8. A person who is currently living a successful life will continue to stay successful. (CH)
9. An individual who is currently honest will stay honest in the future. (CH)
10. The whole, rather than its parts, should be considered in order to understand a phenomenon. (AT)
11. It is more important to pay attention to the whole than its parts. (AT)
12. The whole is greater than the sum of its parts. (AT)

CA = Causality, CO = Attitude towards contradiction, CH = Perception of change, AT = Locus of attention

Syllogistic Reasoning

See Markovitz & Nantel (1989).

Subjective Numeracy Scale

See Fagerlin et al. (2007).

1.2 Study 2

Paranormal Belief and Belief in Pseudoscience Scale

Same as Study 1.

<u>REI-10 (Epstein et al., 1996)</u>

1. I don't like to have to do a lot of thinking. (R)
2. I try to avoid situations that require thinking in depth about something. (R)
3. I prefer to do something that challenges my thinking abilities rather than something that requires little thought.
4. I prefer complex to simple problems.
5. Thinking hard and for a long time about something gives me little satisfaction. (R)
6. I trust my initial feelings about people.
7. I believe in trusting my hunches.
8. My initial impressions of people are almost always right.
9. When it comes to trusting people, I can usually rely on my "gut feelings".
10. I can usually feel when a person is right or wrong even if I can't explain how I know.

Items 1-5 = rationality subscale, 6-10 = experientiality subscale, (R) = reverse coded items

<u>Dialectical Self Scale</u>

See Spencer-Rogers et al. (2004).

<u>Syllogistic Reasoning, Subjective Numeracy, and CRT</u>

Same as Study 1

1.3 Study 3

<u>Dialectic Thinking Task</u>

1. Precognition (Paranormal)
Pro: Some people can have a dream that has predicted some future event.
Anti: No one can have a dream that has predicted some future events.

2. Telepathy (Paranormal)
Pro: Some people can be in communication with others using telepathy.
Anti: People cannot be in communication with others using telepathy.

3. ESP (Paranormal)
Pro: ESP and/or psychokinesis can be demonstrated in the laboratory.
Anti: ESP and/or psychokinesis cannot be demonstrated in the laboratory.

4. Out of Body Experience (Paranormal)
Pro: Mind or soul can leave our body and travel.
Anti: Mind or soul cannot leave our body and travel.

5. Incantation (Paranormal)
Pro: Some people can cast spells on others through the use of formulas and incantations.
Anti: No one can cast spells on others through the use of formulas and incantations.

6. Homeopathy (Pseudoscience)
Pro: Homeopathic remedies foster spontaneous healing.
Anti: Homeopathic remedies cannot foster spontaneous healing.

7. Game brain (Pseudoscience)
Pro: Playing videogame can damage functions of the prefrontal cortex.
Anti: Playing videogame can enhance functions of the prefrontal cortex.

8. Reiki (Palm) healing (Pseudoscience)
Pro: Reiki healing, otherwise known as Palm healing, is effective in healing the body.
Anti: Reiki healing, otherwise known as Palm healing, is useless in healing the body.

9. Fatty-acid supplements (Pseudoscience)
Pro: Fatty acid supplements (omega-3 and omega-6, such as Docosahexaenoic acid and Linoleic acid) have a positive effect on academic achievement.
Anti: Fatty acid supplements (omega-3 and omega-6, such as Docosahexaenoic acid and Linoleic acid) have no effect on academic achievement.

10. Sugary drinks and snacks (Pseudoscience)
Pro: Children are less attentive after consuming sugary drinks, and/or snacks.
Anti: Children are more attentive after consuming sugary drinks, and/or snacks.

Set A (1P, 2A, 3P, 4A, 5A, 6A, 7P, 8A, 9P, 10A), Set B (1A, 2P, 3A, 4P, 5P, 6P, 7A, 8P, 9A, 10P)

Response option: 1: Strongly Disagree - 4: Neither disagree nor agree - 7: Strongly Agree

<u>REI-10, Syllogistic Reasoning, Subjective Numeracy, CRT</u>

Same as Study 2

<u>Analysis-Holism items (Study 1, Choi et al., 2007)</u>

We adopted 6 Causality and 6 Attitude Toward Contradiction items. See Choi et al. (2007) for details.

1.4 Study 4

CRT

We used original 3-item version (Frederick, 2005)

Analysis-Holism items (Study 1, Choi et al., 2007)

We used full 24-items from Choi et al. (2007)

Modified Snowy Pictures Task

See Whitson & Galinsky (2008).

Profundity Judgment Task

See Pennycook et al. (2015).
